# Osteoprotegerin mediates tumor-promoting effects of Interleukin-1beta in breast cancer cells

**DOI:** 10.1186/s12943-017-0606-y

**Published:** 2017-02-01

**Authors:** Stephanie Tsang Mui Chung, Dirk Geerts, Kim Roseman, Ashleigh Renaud, Linda Connelly

**Affiliations:** 1 Department of Pharmaceutical Sciences, Daniel K. Inouye College of Pharmacy, University of Hawaii at Hilo, Hilo, Hawaii USA; 2 Department of Pediatric Oncology, Erasmus University Medical Center, Rotterdam, The Netherlands

**Keywords:** Breast cancer, Inflammation, Interleukin-1beta, Metastasis, Osteoprotegerin, p38 MAPK, p42/44 MAPK

## Abstract

**Background:**

It is widely recognized that inflammation promotes breast cancer invasion and metastasis. Given the complex nature of the breast tumor inflammatory microenvironment, much remains to be understood of the molecular mechanisms that govern these effects. We have previously shown that osteoprotegerin knockdown in breast cancer cells resulted in reduced invasion and metastasis. Here we present novel insight into the role of osteoprotegerin in inflammation-driven tumor progression in breast cancer by investigating the link between osteoprotegerin, macrophages and the potent pro-inflammatory cytokine Interleukin-1beta.

**Methods:**

We used human breast cancer cell lines to investigate the effects of Interleukin-1beta treatment on osteoprotegerin secretion as measured by ELISA. We analyzed public datasets containing human breast cancer genome-wide mRNA expression data to reveal a significant and positive correlation between osteoprotegerin mRNA expression and the mRNA expression of Interleukin-1beta and of monocyte chemoattractant protein CC-chemokine ligand 2. Osteoprotegerin, Interleukin-1beta and CC-chemokine ligand 2 mRNA levels were also examined by qPCR on cDNA from normal and cancerous human breast tissue. We determined the effect of Interleukin-1beta–producing macrophages on osteoprotegerin expression by co-culturing breast cancer cells and differentiated THP-1 macrophages. Immunohistochemistry was performed on human breast tumor tissue microarrays to assess macrophage infiltration and osteoprotegerin expression. To demonstrate that osteoprotegerin mediated functional effects of Interleukin-1beta we performed cell invasion studies with control and OPG siRNA knockdown on Interleukin-1beta-treated breast cancer cells.

**Results:**

We report that Interleukin-1beta induces osteoprotegerin secretion, independent of breast cancer subtype and basal osteoprotegerin levels. Co-culture of breast cancer cells with Interleukin-1beta-secreting macrophages resulted in a similar increase in osteoprotegerin secretion in breast cancer cells as Interleukin-1beta treatment. Macrophage infiltration correlates with osteoprotegerin secretion in human breast tumor tissue samples. We show that osteoprotegerin secretion is regulated by Interleukin-1beta in a p38- and p42/44-dependent manner. We also demonstrate that osteoprotegerin knockdown represses Interleukin-1beta expression, Interleukin-1beta-mediated breast cancer cell invasion and MMP3 expression.

**Conclusions:**

These data indicate a novel role for osteoprotegerin as a mediator of inflammation- promoted breast cancer progression.

**Electronic supplementary material:**

The online version of this article (doi:10.1186/s12943-017-0606-y) contains supplementary material, which is available to authorized users.

## Background

The association between inflammation and cancer development has recently received wide recognition as a cancer hallmark [1]. Persistent inflammation contributes to the progression of cancer by promoting tumor survival, proliferation, angiogenesis, metastasis, and immune evasion [2]. The processes involved in cancer-associated inflammation entail complex interactions between the tumor cells and the tumor microenvironment. The key mediators include inflammatory cells and pro-inflammatory cytokines. Inflammatory cells, in particular tumor-associated macrophages (TAMs), are present in most malignant tumors, and high TAM density is correlated with poor prognosis [2–4]. Amongst the cells in the tumor microenvironment and tumor cells themselves, TAMs serve as a major source of pro-inflammatory cytokines that influence tumor progression [3]. Abundant at tumor sites, IL-1 is one of the most potent pro-inflammatory cytokines that can modulate the growth and invasive properties of tumor cells [3]. IL-1 exists in two agonistic forms, IL-1alpha and -beta (IL1B). IL1B is active as a secreted form whereas IL-1alpha is active as an intracellular protein. Elevated IL1B levels in tumor and serum are associated with higher tumor grade and increased invasion in breast and pancreatic cancer and in myelogenous leukemia, and are correlated with poor patient outcome [5–10]. Ultimately, the primary cause of mortality from cancer is due to metastasis, the spreading of primary tumor cells to distant sites to form secondary tumors. IL1B has been shown to play a significant role on tumor invasiveness and metastasis progression [9, 11–13]. Studies in vivo showed reduced hepatic and lung metastasis of B16 melanoma cell xenografts in IL1B knockout mice [11, 12]. In human breast carcinoma tissues, IL1B levels were found elevated in higher grade tumors [14] and in invasive breast carcinoma versus ductal carcinoma in situ (DCIS) and benign lesions [5].

IL1B has been shown to up-regulate Osteoprotegerin (OPG) expression in the breast cancer cell lines MCF-7 and MDA-MB-231 [15]. OPG is a secreted member of the tumor necrosis factor (TNF) receptor super-family, prominently known for its role as a decoy receptor in bone resorption *in vivo,* and for its inhibition of TNF-related apoptosis-inducing ligand (TRAIL) mediated apoptosis in vitro [16, 17]. There is increasing evidence for a role of OPG in cancer, as OPG expression has been found elevated in more aggressive solid tumors [18–21]. A number of studies support a tumor-promoting effect of OPG in breast cancer [22]. OPG over-expression in MCF-7 (estrogen receptor, ER+) breast cancer cells resulted in increased tumor growth and osteolysis in mouse xenografts [23]. Recently, we reported that siRNA-mediated OPG knockdown in triple-negative breast cancer cells reduced invasion and metastasis in a chick embryo in vivo model [24]. Based on these findings we hypothesized that IL1B modulates breast cancer invasion and metastasis by OPG regulation.

Breast cancer metastasis poses significant treatment challenges. Furthering our understanding of the molecular processes involved is essential for novel therapeutic strategies for metastatic breast cancer. In this current study, we investigate the IL1B-mediated upstream signaling events involved in OPG expression, look into the involvement of macrophages in OPG expression, and examine the link between OPG and IL1B as a novel inflammatory pathway promoting breast cancer metastasis.

## Methods

### Reagents and cell culture

Recombinant human IL1B (200-01B) and IL-1R antagonist (IL1-RA, 200-01R) were purchased from Peprotech (Rocky Hill, NJ). p38 MAPK (8690), phospho-p38 MAPK (Thr180/Tyr182; 4511), p42/44 MAPK (9107S), phospho-p42/44 MAPK (Thr202/Tyr204; 9101) antibodies were purchased from Cell Signaling Technology (Beverly, MA). MAPK inhibitors SP600125, SB202190 and SB203580 were purchased from Sigma Aldrich (St Louis, MO), U0126 and BAY869766 were purchased from Santa Cruz Biotechnologies (Santa Cruz, CA).

The human breast cancer lines: MDA-MB-231, MDA-MB-436, BT549, SKBR3, ZR75-1, HCC1954 were cultured in Dulbecco’s Modified Eagle’s medium (DMEM) supplemented with 10% fetal bovine serum (FBS; Atlanta Biologicals, Lawrenceville, GA), 2 mM L-glutamine, and 50 μg/mL gentamicin (Life Technologies, Carlsbad, CA). THP-1 monocyte cells were cultured in RPMI 1640 supplemented with 10% FBS, 2 mM L-glutamine, 1 mM sodium pyruvate, 10 mM HEPES, and 1% antibiotic/antimycotic solution (15240062, Life Technologies). All cell lines were recently acquired from the ATCC (Manassas, VA). Cell lines were incubated in a humidified atmosphere of 5% CO_2_ at 37 °C.

### Enzyme-linked immunosorbent assay

5 × 10^5^ breast cancer cells were seeded in 2 mL of medium in a 6 well plate and incubated for 48 h. Treatment with IL1B or IL-1RA was administered for the last 24 h. OPG protein from cell culture supernatant was measured using the OPG/TNFRSF11B DuoSet (R&D Systems, Minneapolis, MN). IL1B protein from cell culture supernatant was measured using the Human IL1B ABTS ELISA Development Kit (Peprotech).

### Western blot

Protein extracts were obtained by cell lysis in M-PER (Pierce Biotechnology, Rockford, IL) and Halt protease inhibitor cocktail (Pierce Biotechnology). Proteins were separated by SDS-PAGE and blotted onto nitrocellulose membranes. Membranes were blocked with Blocking Buffer (LI-COR, Lincoln, NE) and incubated with specific antibodies. Protein signals were visualized using the Odyssey infra-red imaging scanner and software (LI-COR).

### Real-time polymerase chain reaction

Total RNA was extracted from breast cancer cells using the RNeasy kit (Qiagen, Germantown, MD). cDNA was prepared from RNA (200 ng) in a 20 μL reaction using the iScript cDNA synthesis kit (Biorad, Hercules, CA). qRT-PCR reactions were performed in 25 μL mixtures containing 1 μL of cDNA, 2x iQ SYBR Green supermix (Biorad), and forward and reverse primers. cDNA’s diluted 1:3 were used for the qRT-PCR reactions of the 18S rRNA housekeeping gene. See Additional file 1: Table S1 for primer sequences. mRNA was normalized to 18S rRNA. Relative expression was determined by the ΔΔCT method [25].

### siRNA transfection

Breast cancer cells were transfected with OPG Stealth RNA siRNA (Stealth siRNAs for human OPG: HSS107349 [#1], HSS181651 [#2] and HSS181652 [#3]) or Negative Control Medium GC siRNA using Lipofectamine RNAiMax, according to the manufacturer’s instructions (all from Life Technologies).

### Transwell co-culture assay

On Day 0, 1 ×10^6^ THP-1 cells/well were seeded into a 6 well plate. On Day 1, the THP-1 cells were treated with 1 mM PMA to induce macrophage differentiation, and 5 × 10^5^ breast cancer cells were seeded onto polycarbonate inserts for 6-well plates (pore size 0.4 μm; Corning Costar, Tewksbury, MA). On Day 2, 4 mL of fresh THP-1 medium was placed onto the THP-1 cells prior to the transfer of the transwell inserts containing the breast cancer cells. The co-cultures were incubated for 8 h in a humidified atmosphere of 5% CO_2_ at 37 °C.

### Invasion assay

MDA-MB-436 cells were transfected with OPG or negative control siRNA as described above. Twenty-four hours post-transfection, cells were treated with IL1B (10 ng/mL) in medium containing 0.5% FBS for another 24 h. Following treatment, cells were seeded at 5 × 10^4^ cells/well into a 96 well cell invasion chamber plate pre-coated with 1x Collagen IV using the Cultrex 96 Well Collagen IV Cell Invasion Assay (3458-096-K, Trevigen, Gaithersburg, MD),. The invasion assay was incubated for 24 h and analyzed according to the manufacturer’s instructions.

### Public dataset analysis

Human breast cancer genome-wide mRNA expression datasets from patient sample series deposited for public access in a MIAME-compliant format were obtained through the Gene Expression Omnibus (GEO) database at the NCBI website (http://www.ncbi.nlm.nih.gov/geo/), except for the Chin-124 set (E-TABM-158) from EMBL/EBI (http://www.ebi.ac.uk/arrayexpress), and two TCGA sets from https://gdc-portal.nci.nih.gov/. Datasets were analyzed using R2; a genomics analysis and visualization platform developed in the Department of Oncogenomics, Academic Medical Center, Amsterdam, The Netherlands (http://r2.amc.nl/). Expression data were uploaded into R2 and analyzed as described previously [26]. Briefly, gene transcript levels from Affymetrix array studies were determined from data image files using GeneChip operating software MAS5.0 and GCOS1.0, from Affymetrix (Santa Clara, CA). Samples were scaled by setting average intensity of the middle 96% of probe signals to a fixed value of 100 for every sample in the dataset, allowing comparisons between micro-arrays. The Illumina arrays for Servant-343 and Jonsdottir-94, and the Agilent arrays for TCGA-528 and −1097 underwent custom processing and normalization as described on their websites. All 29 public datasets were scrutinized for OPG (TNFRSF11B gene) mRNA expression and OPG-correlating genes. 13 datasets with a sample size < 100 and/or the absence of OPG mRNA expression or correlating genes were omitted from further analysis: Black-107 (GSE36771), Clynes-121 (GSE42568), Concha-66 (GSE29431), Desmedt-55 (GSE16391), Jonsdottir-94 (GSE46563), Loi-77 (GSE9195), Miller-116 (GSE5462), Minn-96 (GSE2603), Prat-156 (GSE50948), Quiles-61 (28844), Sotiriou-120 (GSE16446), Sotiriou-198 (GSE7390), and Wessels-60 (GSE41656). The TranscriptView tool (http://bioinfo.amc.uva.nl/human-genetics/transcriptview/) was used to select probe-sets for the Affymetrix and Illumina datasets. No sequence data were available for the two TCGA datasets. Probes had to show unique mapping in an anti-sense position within (late) coding exons and/or the 3’ UTR of the gene. When multiple correct probe-sets were available for a gene, the probe-set with the highest average expression and the highest amount of present calls for that dataset were used. All probe-sets used meet these criteria. In no cases did additional probe-sets show a conflicting result for that dataset.

### Gene expression analysis of human breast cancer and normal tissues

qPCR was performed to characterize the OPG, IL1B and CCL2 mRNA expression profiles using the TissueScan Breast Tissue qPCR Array (OriGene, Technologies, Rockville MD) containing cDNA from normal and breast cancer tissue from different disease stages. See Additional file 1: Table S1 for primer sequences. Data were presented as delta Ct values normalized against β-actin.

### Immunohistochemistry

Human breast cancer tissue microarray slides (Abcam, Cambridge, MA) were separately immunostained for OPG (Abcam) and CD68 (Dako North America, Inc. Carpinteria, CA). Following deparaffinization in xylene and rehydration, slides were subjected to antigen retrieval (10 mmol/L citrate buffer; pH 6.0), followed by 3% hydrogen peroxide incubation, and blocking in 1.5% goat serum. Slides were then incubated with diluted primary antibody followed by incubation with HiDef amplifier secondary antibody, and detection with HiDef polymer (Cell Marque Laboratories, Rocklin, CA) and DAB substrate (3,3-diaminobenzidine; Cell Marque Laboratories). Counterstaining was performed with hematoxylin. The tissue sections were scored semi-quantitatively by a pathologist based on staining intensity. Staining and analyses were performed by the University of Hawaii Cancer Center-Pathology Shared Resource.

### Statistical analysis

Results are presented as mean ± SD. Statistical analyses were determined using a Student’s *t*-test, as indicated in the text. Analyses were performed and graphs were plotted in Prism V6 (GraphPad Software). Correlations between OPG and CCL2/IL1B mRNA expression (Fig. 4) were calculated using R2. Briefly, a Pearson test was performed on 2log-transformed expression values (with the significance of a correlation determined by t = r/sqrt((1-r^2)/(n-2)), where r is the correlation value and n is the number of samples, and distribution measure is approximately as t with n-2° of freedom). Correlations were only calculated for datasets when ≥ 10% of samples had a present call for both genes. For all statistical analyses, *p* < 0.05 was considered significant.

## Results

### IL1B induces OPG expression in different subtypes of breast cancer cells

**Fig. 1:**
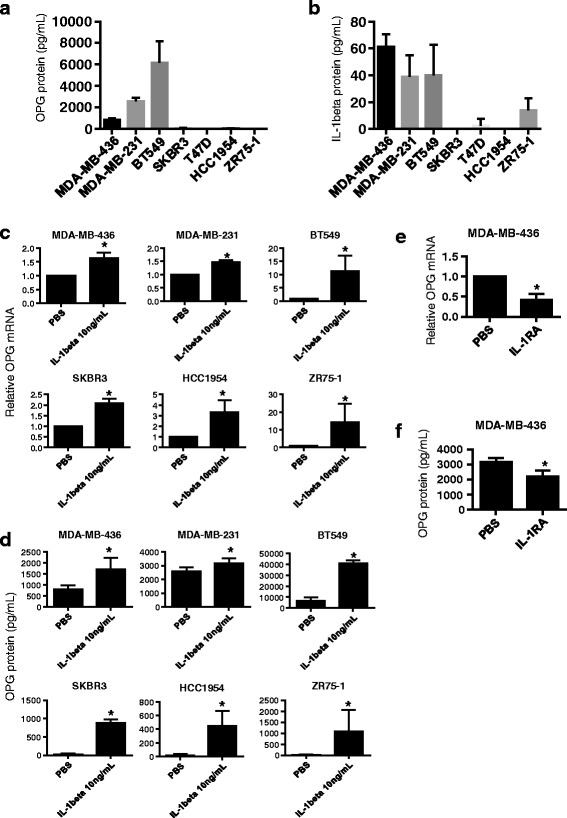
IL1B induces OPG secretion in breast cancer cell lines regardless of subtype and basal OPG protein levels. **a** Basal OPG and **b** IL1B protein levels were assessed by ELISA on supernatant collected from several different breast cancer cell lines, (*n* ≥ 3). **c** Relative OPG mRNA measured by qRT-PCR and **d** OPG secreted protein levels increase upon treatment with IL1B (10 ng/mL) in several different breast cancer cell lines, (*n* ≥ 3). **e** OPG RNA and **f** OPG secreted protein levels decrease upon treatment with PBS or IL-1B receptor antagonist IL-1RA (50 ng/mL) of MDA-MB-436 cells (*n* = 4). Data are represented by mean ± SD. Asterisks indicate statistical significance (*p* < 0.05)

Basal levels of OPG and IL1B protein were examined across cell lines representing multiple breast cancer subtypes. Triple-negative breast cancer (TNBC) cells, the most aggressive breast cancer subtype, lack ER and progesterone receptor (PR) expression, and do not show expression or amplification of the Her2/neu receptor. The TNBC cell lines included in this study, MDA-MB-436, MDA-MB-231 and BT549, were found to secrete higher basal OPG levels than the non-TNBC cell lines T47D, ZR75-1 (both ER+), HCC1954, or SKBR3 (both HER2+; Fig. 1a). Elevated IL1B levels are associated with poor prognosis in cancer, including breast cancer [27, 28]. A study that examined human tissue extracts reported higher IL1B levels in invasive breast carcinoma compared to non-invasive breast tumors [5]. As seen in Fig. 1b, the TNBC lines secreted higher basal IL1B levels compared to the non-TNBC lines. Exogenous IL1B can induce OPG expression in human breast cancer and other cancer cell lines [15, 19, 29]. We therefore asked whether breast cancer cells with higher basal OPG and IL1B secretion levels show different responses to IL1B-induced OPG expression. To test this, breast cancer cells were treated with IL1B (10 ng/mL) for 24 h. Regardless of the basal OPG/IL1B levels, exogenous IL1B significantly induced both OPG mRNA (Fig. 1c) and OPG secretion (Fig. 1d). Notably, cell lines with very low to undetectable basal OPG protein levels, e.g. the non-TNBC cell lines SKBR3, HCC1954 and ZR75-1, showed the highest IL1B-mediated induction of OPG secretion (SKBR3, 880.0 ± 48.7; HCC1954, 448.0 ± 88.2; ZR75-1, 1653.2 ± 927.7; data presented as mean ± S.D. pg/mL). In these cells, IL1B could induce OPG levels comparable to the basal levels of the TNBC MDA-MB-436 cells (805.9 ± 185.6 pg/mL; Fig. 1d).

TNBC-type cell lines exhibited the highest basal OPG and IL1B secretion levels. To evaluate whether basal IL1B influences the basal OPG levels also in these cells, we treated MDA-MB-436 cells with an IL-1B receptor antagonist (IL-1RA, 50 ng/mL) for 48 h. We observed that IL-1RA treatment significantly lowered the OPG mRNA and secreted protein levels (Fig. 1e and f). This finding, along with the observed correlated elevation of OPG and IL1B in TNBCs (Fig. 1a and b), suggests an IL1B autocrine loop for OPG production that may be characteristic of breast tumor progression. Overall, our data shows OPG can be induced across different breast cancer subtypes independent of basal OPG and IL1B secretion levels.

### IL1B upregulates OPG expression by activation of the p38 and p42/44 MAPK signaling pathway

**Fig. 2:**
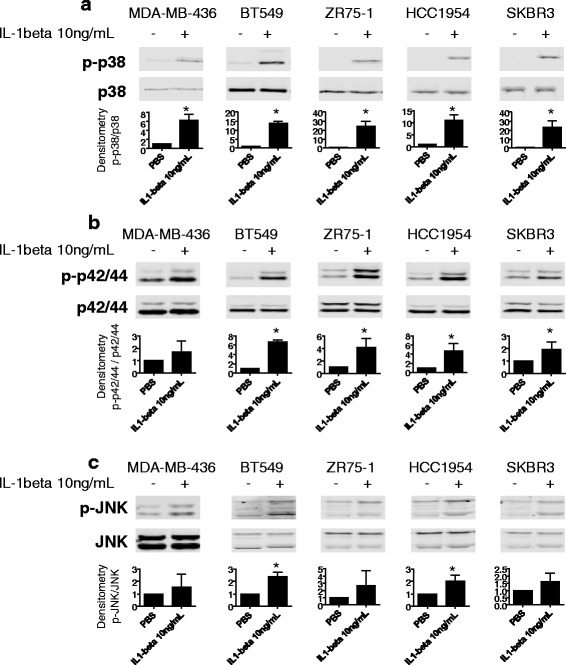
IL1B induces p38, p42/44 and JNK phosphorylation in breast cancer cell lines. Western blot analysis showing **a** p38 **b** p42/44 and **c** JNK phosphorylation increases after 30 min treatment with IL1B (10 ng/mL) in breast cancer cell lines. Densitometry analysis shown below each respective blot represents fold change of phosphorylated protein normalized to the respective total protein, relative to the control. Data are represented by mean ± SD. Asterisks indicate statistical significance (*p* < 0.05)

**Fig. 3:**
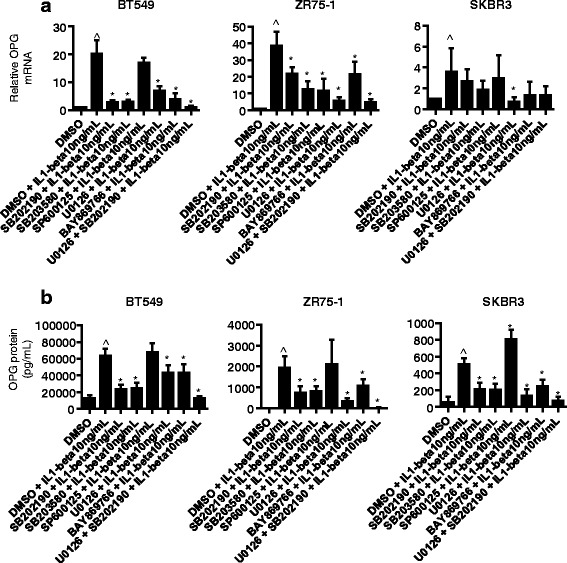
p38 and p42/44 MAPK signaling pathways regulate IL1-mediated OPG secretion in breast cancer cells. **a** Relative OPG mRNA and **b** OPG secreted protein levels measured 24 h after the simultaneous treatment of IL1B (10 ng/mL) and MAPK inhibitor(s) (10 μM) of p38 (SB202190, SB203580), p42/44 (U0126, BAY869766), and JNK (SP600125)) (*n* ≥ 3) in BT549, ZR75-1 and SKBR3 breast cancer cell lines. Data are represented by mean ± SD. ^ denotes *p* < 0.05, as compared to DMSO; asterisks denotes *p* < 0.05, as compared to DMSO + IL1B

The metastatic potential of cancer cells, including breast cancer cells, can be enhanced through mitogen-activated protein kinase (MAPK) signaling [30–35]. To explore the potential involvement of MAPK activation in IL1B-mediated OPG secretion, we first assessed the effects of IL1B treatment on phosphorylation of the p38, p42/44 and JNK MAPK pathway kinases by Western blot and densitometry analysis as shown in Fig. 2. IL1B treatment resulted in increased phosphorylation as compared to untreated controls in all five breast cancer cell lines tested for p38, and in four out of five cell lines tested for p42/44. While IL1B treatment also showed a pattern of increased JNK phosphorylation, the increase was only significantly different from untreated cells in two out of the five cell lines tested. We next tested the effects of the p38 (SB203580 and SB202190), p42/44 (U0126 and BAY869766) and JNK (SP600125) inhibitors in three breast cancer cells lines representative of the three subtypes (BT549, ZR75-1 and SKBR3). Breast cancer cells were simultaneously treated with MAPK inhibitors (10 μM) and IL1B (10 ng/mL) for 24 h. We observed that the p38 and p42/44 inhibitors repressed the IL1B-mediated induction of OPG mRNA (Fig. 3a) and secreted OPG protein (Fig. 3b) in all three breast cancer cell lines tested. In contrast, treatment with JNK inhibitor did not reduce OPG mRNA and OPG secreted protein levels. Given that the p38 and p42/44 signaling pathways significantly repressed the IL1B-mediated OPG secretion, we next explored the effects of dual inhibition. Breast cancer cells were treated with both p38 (SB202190) and p42/44 (U0126) MAPK inhibitors simultaneously with IL1B for 24 h. Dual inhibition resulted in further repression of IL1B-mediated OPG secretion to levels lower than the respective single inhibitor treatments and comparable to basal levels (Fig. 3b). These results suggest the p38 and p42/44 MAPK pathways play important roles in the IL1B-mediated OPG up-regulation in breast cancer cells.

### High OPG mRNA expression correlates to high IL1B and CCL2 mRNA expression in human breast cancer samples

The results above clearly show a role for IL1B in increasing OPG secretion in breast cancer cell lines in vitro (Fig. 1a-d). To ascertain that these experiments reflect the in vivo situation, in human breast tumors, we performed data mining on publicly available human breast cancer mRNA expression datasets derived from patient samples. We were able to analyze 13 different public datasets (for details see Methods). We found that OPG mRNA expression significantly, positively correlated with IL1B mRNA expression in 11 out of 13 datasets (Table 1), in agreement with what we observed for OPG and IL1B secretion in the breast cancer cell lines in vitro. Since the results were so consistent, over 11 of 13 datasets, which had very different tumor subtype and grade compositions, contained patients from diverse geographical regions, and were analyzed on four separate array platforms, we propose that these results are very robust. OPG mRNA expression was also found to correlate with CC-chemokine ligand 2 (CCL2) in all 13 datasets (Table 1).

**Table 1:** OPG mRNA expression is significantly correlated to CCL2 and IL1B mRNA expression in breast cancer

Dataset	CCL2		IL1B	
	*R*	*P*	*R*	*P*
Bertucci - 266	0.269	8.8 • 10^−6^	0.177	3.8 • 10^−3^
Bos - 204	0.330	1.4 • 10^−6^	0.193	5.8 • 10^−3^
Chin-124	0.232	9.5 • 10^−3^	0.207	0.02
EXPO Breast - 351	0.153	4.2 • 10^−3^	0.200	1.6 • 10^−4^
Halfwerk - 947	0.077	0.02	n.s.	
Iglehart-123	0.227	0.01	0.200	0.03
Miller-251	0.150	0.02	n.s.	
Servant - 343	0.199	2.0 • 10^−4^	0.252	2.4 • 10^−6^
Smid - 210	0.312	4.0 • 10^−6^	0.198	4.0 • 10^−3^
TCGA Breast - 528	0.236	4.3 • 10^−8^	0.197	5.3 • 10^−6^
TCGA Breast - 1097	0.210	2.3 • 10^−12^	0.253	1.9 • 10^−17^
Wang - 286	0.212	3.1 • 10^−4^	0.165	5.1 • 10^−3^
Zhang - 136	0.293	5.3 • 10^−4^	0.295	5.0 • 10^−4^
	13 of 13		11 of 13	

OPG and CCL2/IL1B mRNA expression and correlation were analyzed in 13 human breast cancer datasets (See Materials and Methods). Columns from left to right: Dataset name (and sample size), OPG-CCL2 correlation (r = correlation, *p* = *p* value; significant and positive in all 13 datasets), and OPG-IL1B correlation (significant and positive in 11 of 13 datasets). Correlations were calculated using a 2log Pearson test

**Fig. 4:**
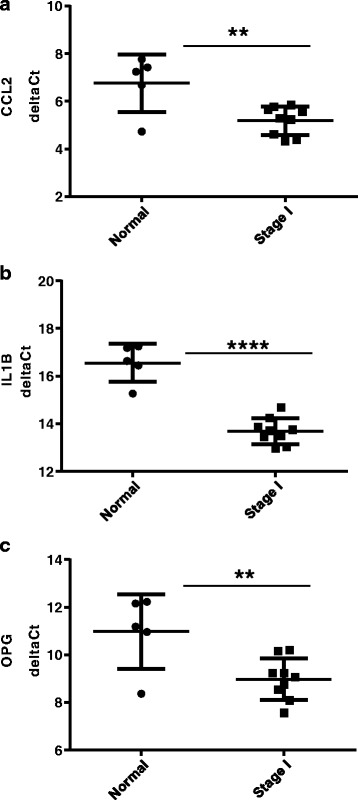
CCL2, IL1B and OPG mRNA levels are elevated in human breast cancer tissue samples. **a** CCL2, **b** IL1B and **c** OPG mRNA levels were measured by qPCR on cDNA samples prepared from normal and breast cancer human tissue samples. Data are represented as delta Ct values after normalizing against β-actin (Ct value for genes of interest minus Ct value of β-actin). Asterisks indicate statistical significance (** *p* ≤ 0.01, ****p ≤ 0.0001)

OPG, IL1B, and CCL2 mRNA in normal and breast cancer human tissue samples were determined by qPCR using a panel of commercially available cDNAs (see Methods). Data was presented as delta Ct values normalized against β-actin (Ct value for genes of interest minus Ct value of β-actin). Higher expression levels are denoted by relative lower delta Ct values and lower levels of expression are denoted by relative higher delta Ct values. We observed statistically significant higher mRNA expression levels (lower delta Ct values) for CCL2 (Fig. 4a), IL1B (Fig. 4b) and OPG (Fig. 4c) in the stage I breast cancer samples relative to normal samples.

The overall patterns observed in the qPCR analysis of a small subset of human breast cancer tissue samples are in agreement with the findings from the public dataset analysis (Table 1) thereby supporting the positive correlation of OPG-CCL2 and OPG-IL1B mRNA expression in breast cancer.

### Macrophage co-culture-induced IL1B elevates OPG expression in breast cancer cells

**Fig. 5:**
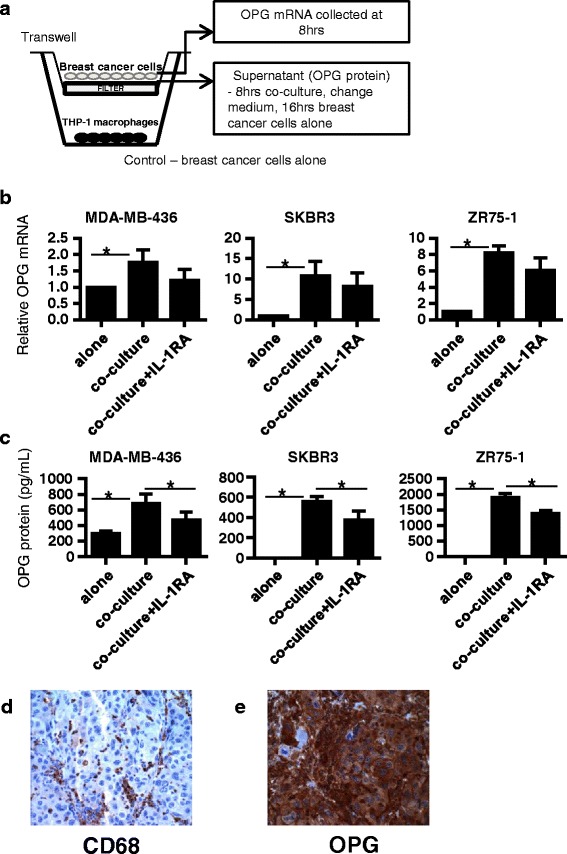
Co-culture with THP-1 macrophages induces OPG secretion in breast cancer cells. **a** Diagram of the transwell co-culture experiment set-up. Breast cancer cells were co-cultured with THP-1 macrophages in the presence and absence of Interleukin-1 receptor antagonist (IL-1RA, 400 ng/mL). **b** After 8 h of co-culture, relative OPG mRNA was measured by qRT-PCR, (*n* ≥ 3). **c** Breast cancer cells were subsequently cultured alone in fresh media for an additional 16 h and OPG secreted protein was measured from this supernatant by ELISA, (*n* ≥ 3). OPG mRNA and secreted protein levels reveal that the co-culture mediated induction of OPG expression in breast cancer cells were partially repressed by IL-1RA. **d** Representative image for **d** CD68 immunohistochemistry and **e** OPG immunohistochemistry on primary/malignant tumor sections on a human breast cancer tissue microarray. Images are taken at 400× magnification. Data are represented by mean ± SD. Asterisks indicate statistical significance (*p* < 0.05)

CCL2 is a potent chemokine involved in the recruitment of monocytes to sites of tissue injury and infection. High breast cancer CCL2 levels are associated with tumor associated macrophage (TAM) infiltration [36]. The mechanisms by which macrophages promote tumor progression are not fully understood. Taking into account the correlation between OPG and CCL2 mRNA expression, we next examined whether macrophages can induce OPG secretion in breast cancer cells. We hypothesized that macrophages could be a source of IL1B that would further stimulate OPG secretion in breast cancer cells. We therefore performed co-culture experiments with macrophages and breast cancer cells representative of different subtypes (MDA-MB-436, SKBR3, and ZR75-1). THP-1 monocytes were seeded and allowed to differentiate to macrophages on the bottom of a 6-well plate before being incubated with a Transwell insert containing breast cancer cells for 8 h (Fig. 5a). After this time point, breast cancer cells were collected for OPG mRNA analysis. In a parallel set up, the Transwell inserts containing breast cancer cells were then moved to fresh media for an additional 16 h, upon which the supernatant was collected for OPG protein analysis. The THP-1 macrophages indeed produced IL1B: 15.0 ± 6.9 pg/mL (mean ± S.D., *n* = 3) of IL1B was detected by ELISA from supernatant taken from THP-1 cells cultured for 8 h. The co-cultures led to significantly increased OPG mRNA and secreted protein in all three breast cancer cells (Fig. 5b and c). To ascertain the specificity of the IL1B-mediated effects, prior to starting the co-culture IL-1RA (400 ng/mL) was added to the media in one of the wells. Under these conditions, the OPG mRNA levels (Fig. 5b), and especially the OPG secreted protein levels (Fig. 5c), were indeed reduced.

Immunohistochemical staining of human primary breast tumors for CD68, a pan-macrophage marker, and OPG revealed that of the 46 samples tested 95.7 and 78.3%, respectively, exhibited moderate/strong staining for these markers (Table 2, Fig. 5d and e). Overall, 76.1% of these samples exhibited moderate/strong crts a link between TAMs and OPG levels in breast tumors.

**Table 2:** CD68 and OPG are commonly co-expressed in primary breast tumors

% with moderate/strong CD68 staining	% with moderate/strong OPG staining	Co-occurrence of moderate/strong CD68 and OPG staining
95.7	78.3	35 out of 46 tumors = 76.1%

CD68 and OPG protein levels were assessed by immunohistochemistry in tissue microarray slides containing human patient primary breast cancer samples (see Methods). Two left columns: data is shown as the percentage of samples presenting with moderate/strong CD68 and OPG staining. Right column: co-occurrence of CD68 and OPG staining represent the detection of moderate/strong expression of both CD68 and OPG within the same patient sample

### OPG is important in the IL1B-enhanced invasion of breast cancer cells

**Fig. 6:**
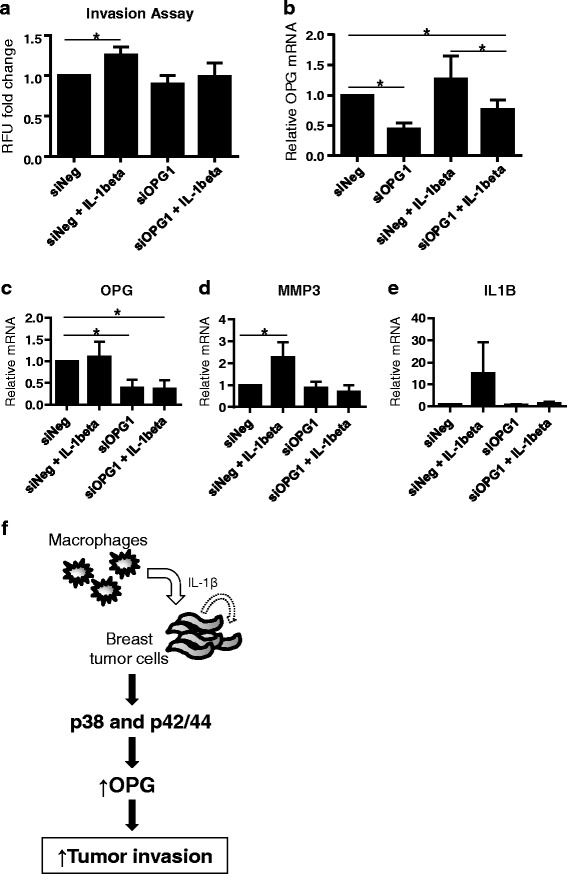
IL1B promotes the invasion of breast cancer cells in an OPG-dependent manner. MDA-MB-436 breast cancer cells transfected with OPG (siOPG1) or negative control (siNeg) siRNA were pretreated with IL1B (10 ng/mL), and subsequently assayed for invasiveness. **a** IL1B-mediated cell invasion is reduced in OPG knockdown cells, (*n* = 3). Data is represented as relative fluorescence units (RFU) fold change relative to the untreated negative control. **b** OPG knockdown in the breast cancer cells used in the cell invasion assay was assessed at the end point of the experiment. Knockdown was verified by qRT-PCR. MDA-MB-436 cells transfected with OPG or control siRNA were treated with IL1B or PBS (10 ng/mL) for 72 h. **c** OPG knockdown at 72 h was verified by qRT-PCR. Assessment of **d** MMP3 and **e** IL1B mRNA levels indicate the IL1B-mediated induction is inhibited by OPG depletion, (*n* = 3). Data are represented by mean ± SD. Asterisks indicate statistical significance (*p* < 0.05). **f** Signaling model. IL1B secreted from macrophages and/or from breast cancer cells themselves, activate p38 and p42/44 MAPK signaling in the breast cancer cells resulting in increased OPG secretion. In turn, this OPG secretion up-regulation promotes the invasiveness of breast cancer cells. OPG levels in breast tumors

A number of studies in breast and prostate cancer, and in melanoma show that tumor cells with increased invasiveness exhibit elevated and autocrine IL-1 production [10, 37]. IL1B has been shown to enhance the invasiveness of TNBC cells in vitro [38, 39] and in IL1B knockout mice, IL1B was essential for the invasiveness of B16 melanoma xenografts [11]. We have recently shown that OPG can promote TNBC cell invasion, so OPG could be a downstream mediator whereby IL1B promotes invasion [24]. To investigate whether OPG indeed plays a role in IL1B tumor-promoting effects, we assessed the effects of OPG knockdown on IL1B-mediated cell invasion. MDA-MB-436 cells were transfected with OPG targeting or control siRNA. Twenty-four hours later, cells were pre-treated with IL1B (10 ng/mL) or PBS in serum reduced medium for 24 h. The invasion assay was carried out for another 24 h. Although not statistically significant, we consistently detected noticeably less invasion of the OPG knockdown cells (Fig. 6a). We also observed that the IL1B-induced cell invasion was significantly elevated in control cells while repressed upon OPG knockdown (Fig. 6a and b).

Matrix metalloproteinases (MMPs) influence cell proliferation, apoptosis and angiogenesis, and play a key role in promoting the invasiveness of tumor cells by cleaving components of the extracellular matrix (ECM) and basement membrane [40]. Han *et al*. reported that MMP-1 and MMP-9 mRNA levels did not differ in TNBC versus non-TNBC cells, but that MMP-3 mRNA levels were higher in TNBC cells [39]. In this same study the authors showed that MMP-3 mRNA and protein were enhanced upon treatment with exogenous IL1B in TNBC cells [39]. In mice, it has been shown that MMP3 over-expression in normal mammary epithelial cells led to oncogenic transformation [41]. We therefore asked whether OPG was important in IL1B-mediated MMP3 induction in breast cancer cells. MDA-MB-436 cells were transfected with OPG targeting or control siRNA. Cells were then treated with IL1B (10 ng/mL) or PBS in serum reduced medium for 72 h prior to collection. Consistent with the previous study, IL1B induced MMP3 mRNA up-regulation in the control cells (Fig. 6d). Upon OPG knockdown, IL1B-mediated MMP3 induction was repressed (Fig. 6c and d). It is known IL1B can induce its own expression, acting in a positive-feedback loop to enhance the IL-1 response [42]. Treatment with exogenous IL1B elevated the IL1B mRNA levels in the breast cancer cells (Fig. 6e). Interestingly, this IL1B-mediated IL1B induction was also repressed upon OPG knockdown (Fig. 6c and e).

Taken together, our findings suggest OPG is a downstream effector of IL1B-mediated invasion in breast cancer cells.

## Discussion

Persistent inflammation is linked with cancer development and progression [43]. It has been shown that treatment with the inflammatory cytokine IL1B promotes the invasiveness of breast cancer cells in vitro [38, 39]. In our previous work we reported that the invasive and metastatic capacity of TNBC cells was reduced upon OPG knockdown [24]. In this study, we investigated OPG in the context of its role in breast cancer and inflammation, with a particular focus on IL1B. Here we provide mechanistic insight into the IL1B-OPG signaling axis and reveal a potential role for OPG in the invasion-promoting effects of IL1B. We show that OPG secretion is induced by IL1B in a p38- and p42/44-dependent manner, independent of breast cancer subtype or basal OPG levels. Macrophages, but possibly also breast cancer cells themselves, may serve as local IL1B sources to influence OPG secretion. Also, we show that IL1B-mediated breast cancer cell invasion, and the induction of MMP3 and IL1B itself, occurs in an OPG-dependent manner.

Elevated OPG secretion has been detected in aggressive tumors with poor patient outcome, including breast, lung, prostate, gastric and bladder cancers [19, 20, 44–46]. In breast cancer cells, OPG over-expression resulted in enhanced tumor growth [23] and increased pulmonary metastasis in mice [47]. In agreement with these reports, we have shown that TNBC cells, representing the most aggressive breast cancer subtype, secreted higher basal OPG levels than non-TNBC cells. Similarly, we showed that TNBC cells secreted higher IL1B levels than non-TNBC cells, which exhibited little to no IL1B secretion. These results are consistent with a study indicating that the non-TNBC cell line HCC1954 produces low IL1B amounts [48]. We found that regardless of the basal OPG and IL1B levels, all breast cancer cell lines remained responsive to IL1B-mediated OPG induction, with the highest induction of OPG secretion in the non-TNBC cells (Fig. 1c and d). Taking into account the association of higher OPG and IL1B basal levels in TNBC cells, we treated the TNBC type MDA-MB-436 cell line with the IL1B receptor antagonist IL-1RA. This resulted in the partial repression of basal OPG secretion suggesting an autocrine loop, by which IL1B produced by cells is linked to the higher basal expression of OPG.

The mitogen-activated protein kinase (MAPK) signaling pathway is often activated in cancer [49, 50]. Indeed, p38 and p42/44 MAPK signaling have been associated with breast cancer invasion and progression. In breast cancer cells it has been reported that elevated p38 MAPK signaling can drive invasiveness and chemoresistance of HER2-overexpressing cells [35]. Another study has reported that patients with lymph node-positive breast carcinoma showed shorter progression-free survival when their primary tumors expressed high levels of phosphorylated p38 [32]. In a study examining primary human breast tumors, 11 out of 23 samples showed active p42/44, significantly elevated relative to adjacent matched normal breast tissue [51]. Interestingly, it has been reported that TNBC, basal-like type breast cancer cell lines, relative to breast cancer cell lines representative of the other subtypes, exhibit a greater sensitivity to p42/44 MEK1/2 inhibitors [52, 53]. In our inhibitor studies we confirmed that both p38 and p42/44 MAPK activities mediated IL1B-induced OPG secretion. This effect was observed independent of breast cancer subtype. Therefore our results show that IL1B-induced OPG secretion is regulated by the p38 and p42/44 MAPK pathways in breast cancer cells.

In an analysis of publicly available human breast cancer genome-wide mRNA expression datasets from patient samples we showed that OPG mRNA expression was significantly correlated with IL1B mRNA expression. This correlation is consistent with in vitro experiments showing that IL1B and OPG levels were either both elevated or both relatively lower in TNBC cells and non-TNBC cells, respectively. CCL2 mRNA expression was also found to be significantly correlated with OPG mRNA expression. CCL2 is a potent chemo-attractant involved in macrophage tissue infiltration. TAMs are present in many solid tumors, including breast cancer. Clinical studies have indicated TAM levels are correlated with breast cancer prognosis [54], and experimental evidence showed that CCL2 levels are significantly associated with TAM numbers [36, 55] and TAM retention [56], implicating CCL2 in breast cancer progression. Furthermore, we showed relative to normal breast tissue that elevated levels of CCL2, IL1B and OPG mRNA levels were detected in stage I breast cancer human tissue samples. We asked whether macrophages could serve as an IL1B source to influence OPG expression in breast cancer cells. Upon co-culture with THP-1 macrophages, OPG was significantly induced in breast cancer cells. This induction was partially repressed in the presence of IL-1RA, indicating that the effects on OPG were specific to IL1B. The potential causative link between macrophages and elevated OPG levels were further supported by the immunohistochemical analyses of pan-macrophage marker CD68 and OPG which indicated a co-occurrence of these two markers in human primary breast tumors. Studies have shown that IL1B and the p38 and p42/44 MAPK pathways play important roles in tumor cell progression [9, 31, 34, 35, 38, 39, 51–53, 57–59]. Given our data demonstrating that OPG is subject to regulation by IL1B-p38 and -p42/44 signaling, we sought to investigate OPG function in IL1B-mediated breast cancer invasion. Our results showed that IL1B treatment significantly elevated the invasiveness of MDA-MB-436 cells. Interestingly, these effects were inhibited upon OPG knockdown. MMPs play key roles in promoting the invasive properties of tumor cells [40]. Particularly, MMP3 has been shown to drive the formation of mammary tumors in mice when over-expressed in mammary epithelial cells [41]. Additionally, MMP3 mRNA levels had been reported to be elevated in TNBC cells versus non-TNBC cells, where treatment with exogenous IL1B could lead to MMP3 up-regulation [39]. In line with this published study, treatment of MDA-MB-436 with IL1B cells induced MMP3 expression. We show IL1B treatment can lead to IL1B up-regulation (Fig. 4d). Induction of both MMP3 and IL1B were repressed in cells treated with OPG siRNA (Figs. 4c-e), this is in agreement with our previous study showing reduced invasion upon OPG knockdown in TNBC cell lines [24]. The effects of OPG knockdown on IL1B induction suggest that IL1B expression may also be subject to regulation by OPG. Further studies are needed to define this cross-regulation between OPG and IL1B. In any case, the interdependent effects of OPG and IL1B expressions further support the significance of OPG in inflammatory-driven tumor progression.

## Conclusions

In summary, we have identified OPG as a potential downstream effector in the metastasis-promoting effects of IL1B in breast cancer. IL1B from macrophages or from breast cancer cells themselves can induce OPG secretion in a p38- and p42/44-dependent manner that contributes to increased invasion (Fig. 6f). The increased invasion promoted by IL1B and OPG involves MMP3 induction as well as an IL1B auto-amplification loop. This study presents a novel pathway whereby inflammation could promote breast cancer progression.

## Additional file

Additional file 1: Primer sequences. (DOCX 11 kb)

## Abbreviations

CCL2: CC-chemokine ligand 2
DCIS: Ductal carcinoma in situ
ECM: Extracellular matrix
ER: Estrogen receptor
IL1B: Interleukin-1beta
IL-1RA: IL-1R antagonist
MAPK: Mitogen-activated protein kinase
MMPs: Matrix metalloproteinases
OPG: Osteoprotegerin
PR: Progesterone receptor
qPCR: quantitative PCR
qRT-PCR: quantitative reverse transcriptase PCR
TAM: Tumor-associated macrophages
TNBC: Triple-negative breast cancer
TNF: Tumor necrosis factor
TRAIL: TNF-related apoptosis-inducing ligand
