# Supplementary material for: Osteoprotegerin mediates tumor-promoting effects of Interleukin-1beta in breast cancer cells
Source: Mol Cancer. 2017 Feb 1;16:27. doi: 10.1186/s12943-017-0606-y (PMC5286681; doi:10.1186/s12943-017-0606-y)
Supplement: Additional file 1: — Primer sequences. (DOCX 11 kb) [file 12943_2017_606_MOESM1_ESM.docx]

**Supplementary data**

**Table S1: Primer sequences**

| 18S rRNA Forward | 5’–GCCCGAGCCGCCTGGATACC–3’ |
| --- | --- |
| 18S rRNA Reverse | 5’–TCACCTCTAGCGGCGCAATACG-3’ |
| OPG Forward | 5’–AACGGCAACACAGCTCACAAGAAC–3’ |
| OPG Reverse | 5’–TGCTCGAAGGTGAGGTTAGCATGT–3’ |
| MMP3 Forward | 5’–CACTCACAGACCTGACTCGG–3’ |
| MMP3 Reverse | 5’–AGTCAGGGGGAGGTCCATAG–3’ |
| IL1B Forward | 5’–CCACCTCCAGGGACAGGATA–3’ |
| IL1B Reverse | 5’–AACACGCAGGACAGGTACAG–3 |
| β-actin Forward | 5’- CAGCCATGTACGTTGCTATCCAGG-3’ |
| β-actin Reverse | 5’- AGGTCCAGACGCAGGATGGCATG-3’ |
| CCL2 Forward | 5’-GGCTGAGACTAACCCAGAAAC-3’ |
| CCL2 Reverse | 5’-GAATGAAGGTGGCTGCTATGA-3’ |
